# Time series strain tracking analysis post fecal transplantation identifies individual specific patterns of fecal dominant donor, recipient, and unrelated microbial strains

**DOI:** 10.1371/journal.pone.0274633

**Published:** 2022-09-15

**Authors:** Hyunmin Koo, Casey D. Morrow

**Affiliations:** 1 Department of Genetics, Hugh Kaul Precision Medicine Institute, University of Alabama at Birmingham, Birmingham, Alabama, United States of America; 2 Department of Cell, Developmental and Integrative Biology, Hugh Kaul Precision Medicine Institute, University of Alabama at Birmingham, Birmingham, Alabama, United States of America; Washington State University - Spokane, UNITED STATES

## Abstract

**Background:**

Fecal microbial transplantation (FMT) has been used with the therapeutic intent to change the functions of the gut microbial community in metabolism and host immunity. For most of these therapies, the recipients are not given antibiotics to eliminate the microbial community prior to transplant with donor fecal microbes resulting in the initial gut microbial community following FMT consisting of a consortium of donor and recipient microbes. The detailed analysis of the fecal samples from these FMT over time provides a unique opportunity to study the changes in the gut microbial strain community that occurs following the introduction of new microbial strains (donor) into an established community (recipient).

**Methods:**

In this study, we have metagenomic data set consisting of 5 FMT that contained donor, recipient and recipient post FMT taken multiple times for periods up to 535 days after the FMT. We used two established strain tracking methods, Window-based Single Nucleotide Variant (SNV) Similarity (WSS) and StrainPhlAn, to determine the presence of donor and recipient microbial strains following FMT. To assess recombination between donor and recipient strains of *Bacteroides vulgatus* post FMT, we used BLAST+ to analyze the data sets for *Bacteroidales*-specific antimicrobial proteins (BSAP-3) that have known functions to restrict species specific replication.

**Results:**

We found that *Alistipes onderdonkii*, *Alistipes shahii*, *Alistipes putredinis*, and *Parabacteroides merdae*, all had patterns post FMT consisting of either dominant donor or recipient microbial strains in the feces. In contrast, the analysis of *Bacteroides spp*. in five FMT pairs revealed inter-individual oscillation over time with the appearance of either donor or recipient fecal strain dominance. In some instances, *B*. *vulgatus* and *B*. *uniformis* were also identified after FMT that were not related to either the donor or recipient. Finally, in one of the FMT, we identified a distinct *B*. *vulgatus* strain post-FMT that matched the pre-FMT strain but was BSAP-3 positive, suggesting a possible recombination event between the donor and recipient strains.

**Conclusion:**

The complex oscillating patterns of the appearance of fecal dominant donor, recipient or unrelated strains following extended times post FMT provide new insights into the dynamics of the microbial community interactions with the recipients following FMT. The result from our analysis has implications for the use of FMT to predictably change the biological functions of the gut community in metabolism and host immunity.

## Introduction

Numerous studies have shown fecal microbial transplant (FMT) to be highly effective for the treatment of patients with recurrent *Clostridium difficile* [1–3]. For these patients, FMT was used primarily as a last resort following the failure of standard therapy that consisted of multiple rounds of suppressive antibiotics that effectively eliminated the recipient microbial community [4–7]. To better characterize the microbial interactions of donor and recipient following FMT, several studies have used metagenomic sequencing coupled with new informatics approaches that allowed the resolution of the microbial community at the strain level [8–11]. We, and others have shown that for *C*. *difficile* patients that have had suppressive antibiotics that FMT can result in the stable long-term colonization of donor microbial strains for up to two years post FMT, the longest time examined [5,8].

FMT has been also used with therapeutic intentions to change the biological functions of the gut microbial community in chronic diseases that impact metabolism and host immunity [12–15]. The analysis of the donor and recipient strains post FMT without antibiotics provides a unique system in which to examine the perturbation of the ecology of the human gastrointestinal microbial community following the introduction of new strains. A study by Li et al. described FMT in which fecal samples from lean patients were used to transplant obese recipients to improve metabolic function [16]. For these experiments, pre-conditioning with antibiotics was not done prior to transplant to reduce the number of recipient microbes. Li et al. found the presence of donor microbial strains in the recipient in some cases up to 84 days post FMT. Furthermore, there was considerable variability between individuals with many times both donor and recipient microbes detected in the same individual FMT [16].

In the current study, we have further investigated the patterns of change in the diversity of donor and recipient microbial strains in recipients following FMT not pre treated with antibiotics using a data set from a recent study that used FMT to enhance immunotherapy using anti-PD-1 [17]. In contrast to Li et al [16], the unique features of this study included multiple longitudinal sampling over periods of time up to 535 days post FMT. The FMT donor-recipient pairs from the Davar et al. study were analyzed for donor and recipient strains in the recipients following FMT using two strain tracking methods [8,10]. Our analysis demonstrates individual FMT specific patterns of the fecal dominant donor or recipient microbial stains following FMT. In some FMT, we also found the strain tracking indicated the presence of microbial strains that were not related to the donor or recipient as well as evidence of at least one instance of possible recombination between the donor and recipient. The presence of these heterogeneous donor-recipient microbial communities post FMT could have an impact on the function of the transplanted microbial community in host metabolism and immune system.

## Materials and methods

### Ethics statement

We obtained the publicly available original sequence files from the NCBI BioProject database under accession number PRJNA672867. All participants’ samples were collected, sequenced, and deposited by Davar et al. [17]. The sequence data that we used in this study was fully anonymized before being made publicly available.

### Data set used in this study

We used publicly available data set, Davar et al. [17], to perform strain-tracking analysis. For this data set, fecal samples from the selected 10 individuals (5 donors and 5 recipients) were selected that provided multiple longitudinal samples. Multiple samples were collected from each donor at various time points and these were merged into a single sample to run the analysis. Pre-FMT (7 to 21 days prior FMT) and longitudinal post-FMT (weekly collected for 12 weeks and then every 3 weeks, if available) samples were collected and sequenced by Davar et al. The data set used in this study were detailed in S1 Table.

### Sequence reads and processing

A total of 1,398,692,232 metagenomic sequencing reads were downloaded from Davar et al. (S1 Table). Due to a difference in read counts between donor and recipient samples, all samples from the 5 donor-recipient pairs were randomly subsampled (seed = 1000) with seqtk (v. 1.3) (https://github.com/lh3/seqtk). The number of subsampled reads for all samples were shown in S1 Table. Quality control steps include filtering short sequences (<50 bases), low quality reads (sliding window 50 bases having a QScore <20) using Trimmomatic (v. 0.36) [18] and removing any human reference genome (hg19) using bowtie2 (v. 2.3.4.3) [19] with default parameters were applied to the subsampled sequence reads.

### Strain profiling using strain-tracking analyses

Taxonomic profiling for all samples was conducted using MetaPhlAn2 [20,21], however only two selected donor-recipient pairs (donor: 18–0014, recipient: 19–0024 and donor: 18–0002, recipient: 18–0018) were shown as figures. MetaPhlAn2 classifies the subsampled sequence reads to taxa and provides their relative abundances in each sample based on the set of marker gene database established in MetaPhlAn2.

Our WSS strain-tracking analysis was conducted for all donor-recipient pairs and the full details of the WSS methods can be found in our previously published papers [8,22–26]. We have additionally performed strain-tracking analysis for *Bacteroides uniformis* for two selected donor-recipient pairs (donor: 18–0014, recipient: 19–0024 and donor: 18–0002, recipient: 18–0018) and for *Bacteroides vulgatus* for three selected donor-recipient pairs (donor: 18–0014, recipient: 19–0024, donor: 18–0002, recipient: 18–0018, and donor: 18–0005, recipient: 18–0007) using StrainPhlAn with default parameters and with the options ‘-relaxed_parameter3, -marker_in_clade 0.1’ [10]. As a complementary approach, we have used StrainPhlAn analysis, which uses SNP-based haplotyping in species-specific marker genes [10]. The resultant file was used to build a phylogenetic tree. The resultant phylogenetic trees for *B*. *uniformis* and *B*. *vulgatus* were visualized using the neighbor-joining method along with the Maximum Composite Likelihood [27] in MEGA X with default parameters [28,29].

### Analysis of *Bacteroides* BSAP-3 gene

The details of the analysis for the genes encoding BSAP-3 can be found in Koo and Morrow [30]. Briefly, before running the analysis, quality control steps were included removing any human reference genome (hg19) using bowtie2 (version 2.3.4.3) with default parameters [19], and filtering low quality reads (sliding window of 50 bases having a QScore <20) using Trimmomatic (version 0.36) [18]. Sequencing reads from donor-recipient pairs were aligned to the BSAP-3 gene from Bacteroides vulgatus CL09T03C04 using Burrows-Wheeler aligner (BWA; version 0.7.13) [31]. Aligned reads from each reference genome were then sorted and indexed using SAMtools (version 0.1.19) [32]. The resultant bam file was converted to FASTQ format using BEDTools (version 2.26.0) [33]. Each converted FASTQ file was then assembled using MEGAHIT and the resultant contig file was selected for BLASTX search against the BSAP-3 sequence read using BLAST+ [34,35]. An FMT pair of donor 18–0005 and recipient 18–0007 was selected for further visualization.

## Results

In a previous study, we have developed a microbial strain tracking analysis called Window-based Single Nucleotide Variant (SNV) Similarity (WSS) program to assess the relatedness of the microbes from two separate fecal samples [8]. Using paired samples (i.e. taken at two different times from the same individual) from the Human Microbiome Project (HMP) data set [36], we also established cutoff values for the WSS scores for microbes that can be used to discern between related and unrelated samples [8]. Thus, for our study, we used WSS strain tracking analysis to analyze the recently described data set where FMT was tested for the capacity to overcome resistance to anti-PD-1 immunotherapy in patients where suppressive antibiotics were not used prior to FMT (detailed description of the patient population can be found in [17]). In 5 of these FMT, an extensive time series of fecal samples were collected post FMT, in some cases up to 535 days, and sequenced by metagenomic DNA sequencing (detailed sample information used in this study listed in S1 Table).

**Fig 1:**
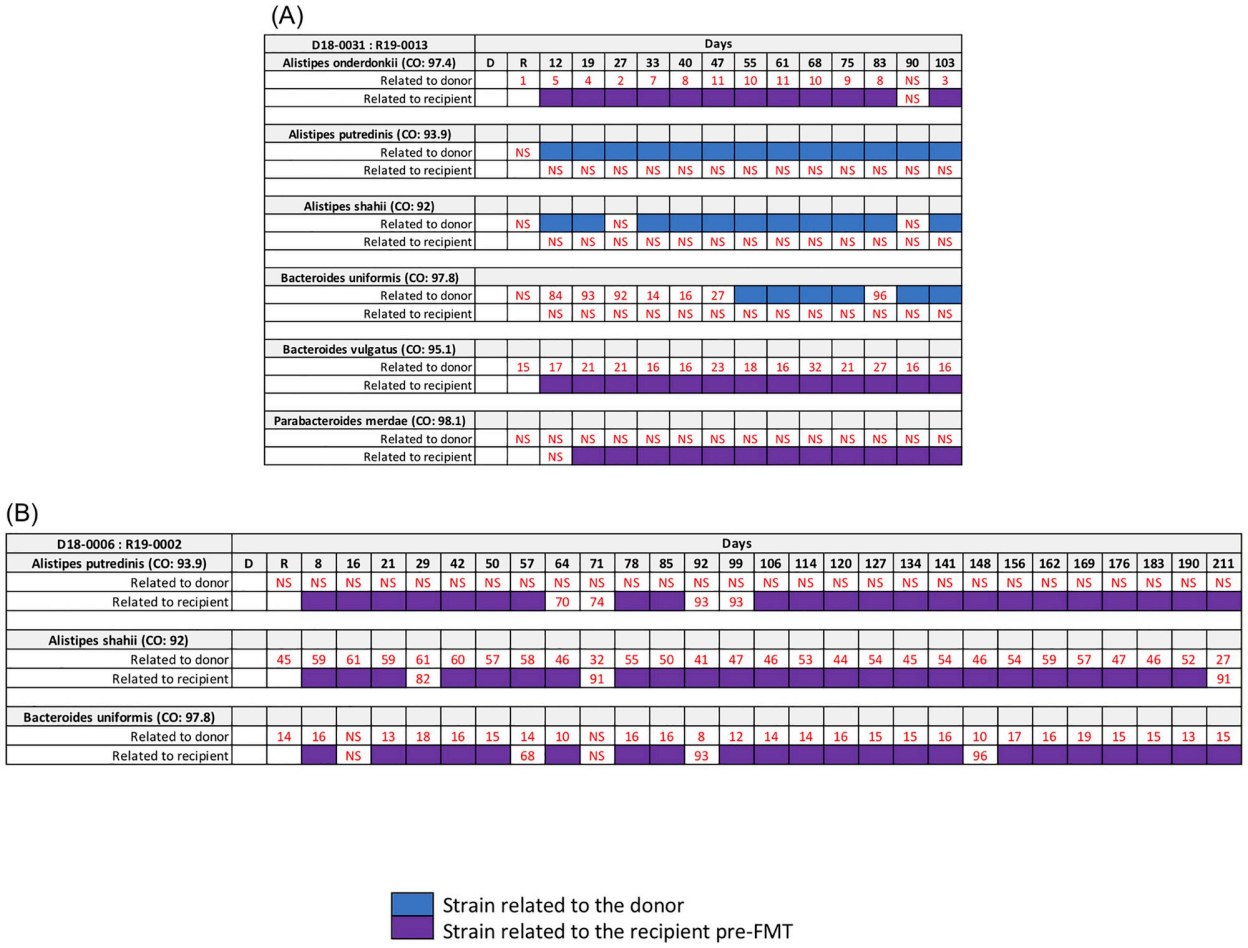
Summarized WSS scores from donor-recipient pairs. The WSS scores were obtained comparing 1) the donor’s sample to paired recipient’s pre- and post-FMT samples; and 2) the recipient’s pre-FMT sample to the same recipient’s post-FMT samples for (A) donor 18–0031 and recipient 19–0013, and (B) donor 18–0006 and recipient 19–0002. All samples used for the analysis were listed in S1 Table. The summarized WSS scores per pair were grouped into different color boxes (see the figure key) or numerical numbers (when observed WSS score was below each species’ cutoff value; NS = No Score). The NS indicates the strains were unable to reliably determine relatedness due to any sample in pairs not satisfying the criteria of WSS analysis (minimum coverage > 30% and average depth > 3.5).

Our first analysis used WSS strain tracking to track donor or recipient strains in the feces following two independent FMT (Fig 1A and 1B). A consistent finding for both FMT was that for most of the microbes examined either donor (blue colored box) or recipient (purple colored box) strain was dominant in the feces of post-FMT as reflected from the WSS score above the cutoff value. An FMT specific pattern with respect to the fecal dominance of either donor or recipient strains was seen for *Alistipes spp*., *Bacteroides spp*., *or Parabacteroides merdae* for the days analyzed post FMT.

**Fig 2:**
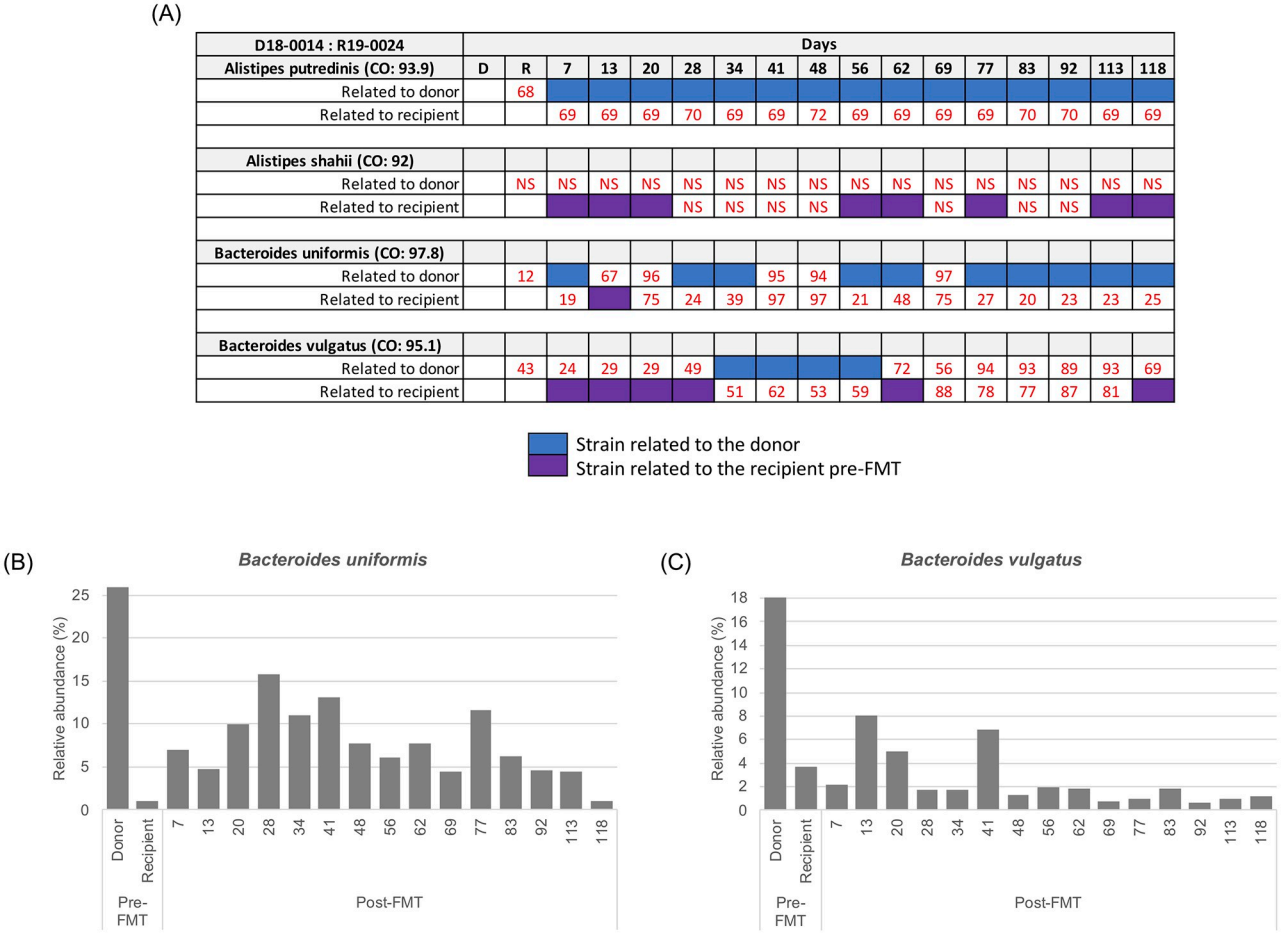
Summarized WSS scores with the taxonomic profile for the donor 18–0014 and recipient 19–0024 FMT pair. (A) The WSS scores were determined by comparing 1) the donor’s sample to paired recipient’s pre- and post-FMT samples; and 2) the recipient’s pre-FMT sample to the same recipient’s post-FMT samples for donor 18–0014 and recipient 19–0024. Detailed information for this pair of samples was shown in S1 Table. The summarized WSS result was grouped into different color boxes (see the figure key) or numerical numbers (when determined WSS score was below the cutoff value; NS = No Score). (B and C) Relative abundance was determined for *Bacteroides uniformis* and *Bacteroides vulgatus* for this pair using MetaPhlAn2 analysis.

We next examined a third FMT (donor 18–0014 and recipient 19–0024) in which samples were collected for 118 days post FMT (Fig 2A). Similar to what we found for the first two FMT, we found from strain tracking that there were strains related to the donor (blue colored box) or the recipient (purple colored box) for the *Alistipes spp*.. In contrast, for the *Bacteroides spp*., we found the presence of both donor and recipient strains after FMT. For example, for *B*. *vulgatus* we found that the recipient was fecal dominant for the first 28 days after the FMT and then the donor microbes were dominant from days 34 to 56. Interestingly, we found for both *B*. *uniformis* and *B*. *vulgatus* that the donor sample had a greater relative abundance than the recipient (Fig 2B and 2C). However, there was no obvious relationship evident between relative abundance and different types of strains post-FMT (Fig 2B and 2C).

**Fig 3:**
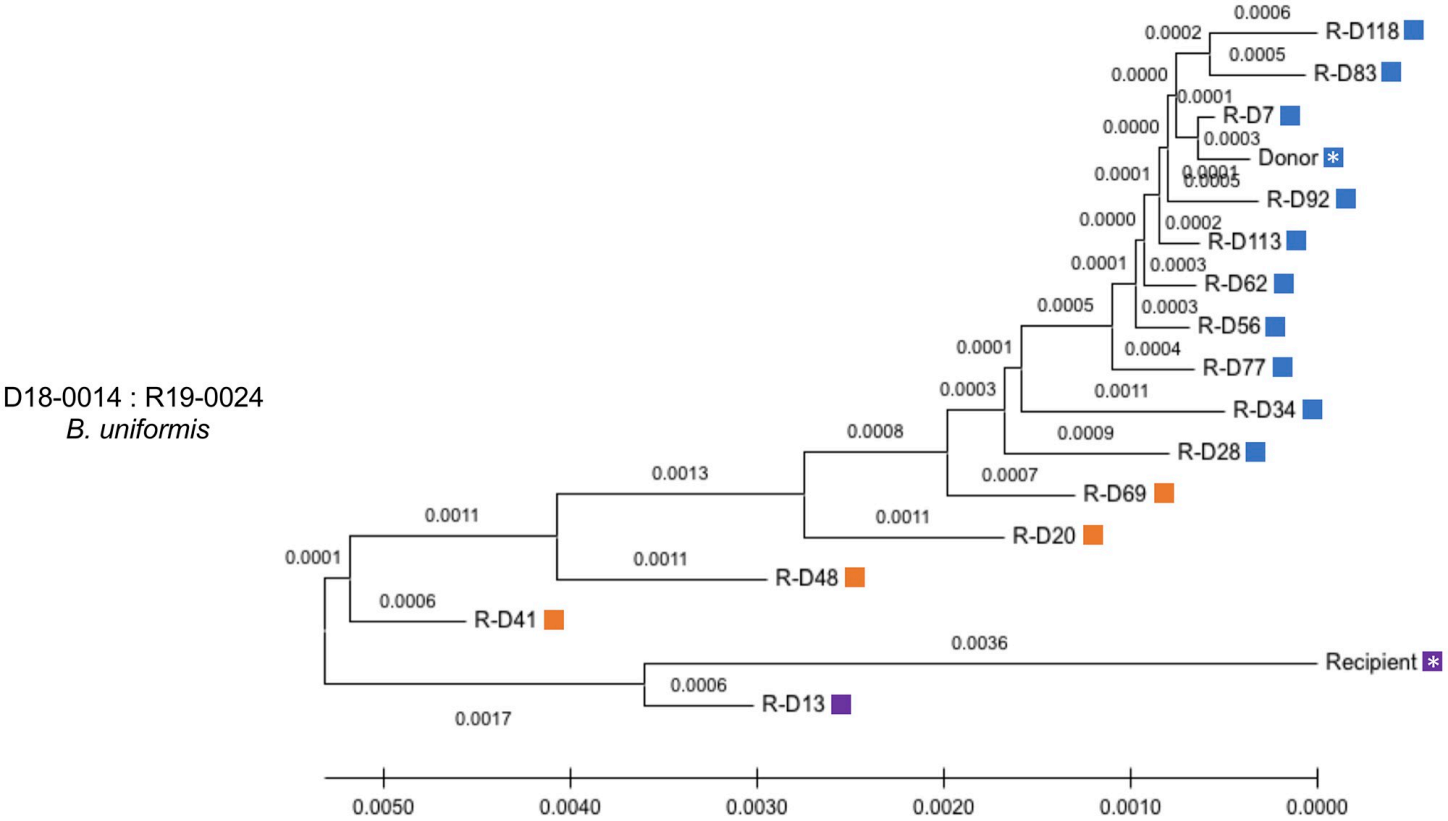
StrainPhlAn analysis for *B*. *uniformis* from FMT donor 18–0014 and recipient 19–0024 pair. StrainPhlAn analysis on FMT pair donor 18–0014 and recipient 19–0024 was done for *B*. *uniformis* across all samples in this pair. A neighbor-joining (NJ) tree was built and the tree is drawn to scale with branch lengths. Then, the distances were calculated using the Maximum Composite Likelihood method and are in the units of the number of base substitutions per site using MEGA X. The blue and purple color boxes shown next to the NJ tree match the color boxes shown in Fig 2A and the orange color boxes indicate a recombinant strain which is shown as both numerical numbers in Fig 2A. A white asterisk indicates a donor and recipient pre FMT sample.

In this FMT, we also found several instances where the WSS scores were high when compared to either donor or recipient. For example, *B*. *uniformis* from days 20, 41, 48, and 69 and *B*. *vulgatus* from days 69, 77, 83, 92, and 113 all had WSS scores higher than the starting WSS score of donor compared to the recipient pre FMT [8]. To further explore this result, we used StrainPhlAn analysis for this pair. From this analysis for *B*. *uniformis*, we found two distinct clusters on the phylogenetic tree one for donor related microbes (blue boxes) and another one for recipient microbes (purple boxes) (Fig 3). Importantly, we found an additional cluster of samples (orange) located in between the donor and recipient, consistent with the WSS scores of samples that were high for both donor and recipient (R-D 41, R-D 48, R-D 20, and R-D 69).

**Fig 4:**
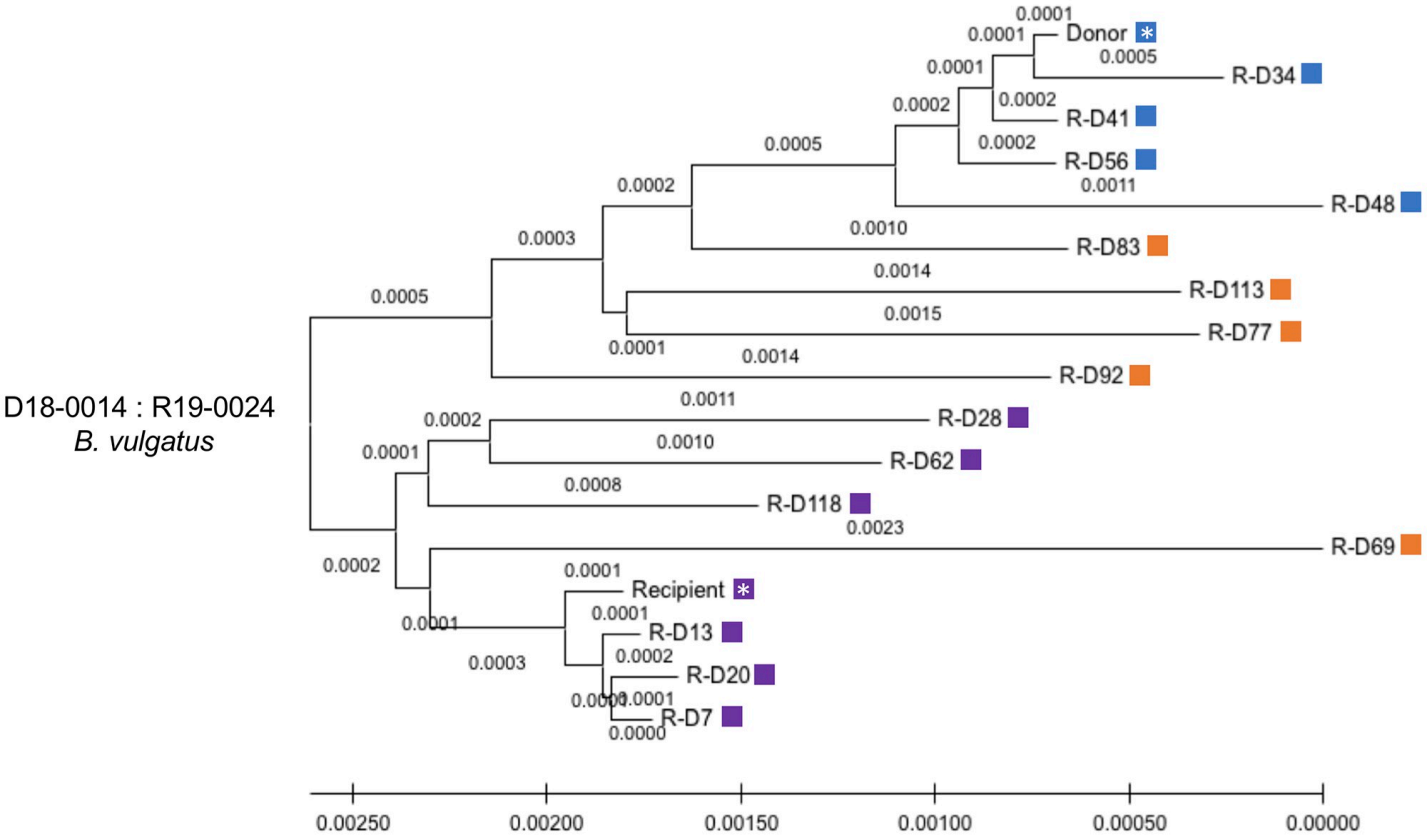
StrainPhlAn analysis for *B*. *vulgatus* on donor 18–0014 and recipient 19–0024 pair. StrainPhlAn analysis on FMT donor 18–0014 and recipient 19–0024 pair was done for *B*. *vulgatus* across all samples in this pair. A neighbor-joining (NJ) tree was constructed and the tree is drawn to scale with branch lengths. Then, the same steps described in the legend of Fig 3 were applied to complete constructing the tree using MEGA X. The blue and purple color boxes shown next to the NJ tree match the color boxes shown in Fig 2A and the orange color boxes show a recombinant strain which is displayed as both numerical numbers in Fig 2A. A white asterisk indicates a donor and recipient pre FMT sample.

A similar pattern was seen in the analysis of the *B*. *vulgatus* where we found many of the samples with high WSS scores related to donor and recipient where the donor and recipient were in separate clusters on the phylogenetic tree (Fig 4). One exception was for sample R-D 69, which was found within a cluster containing the recipient samples. However, this sample had a WSS score that was more related to the recipient (i.e. 88, but still under the cutoff value) than the WSS score related to the donor (i.e. 56).

**Fig 5:**
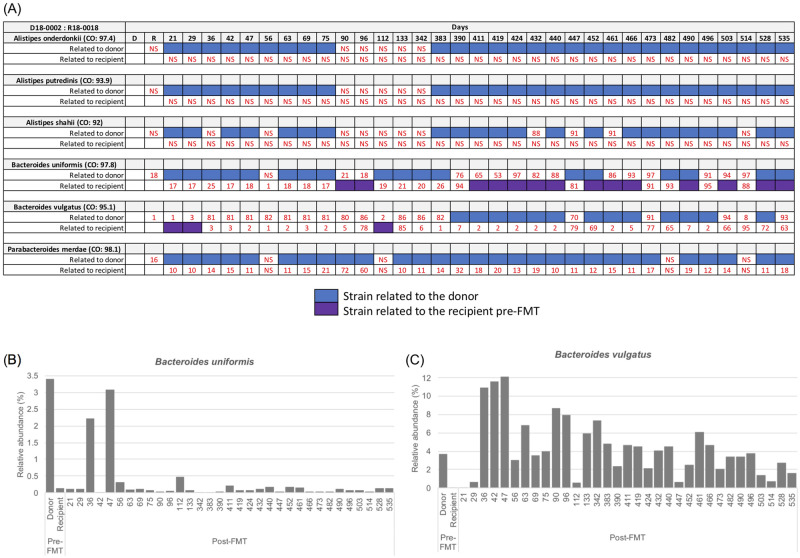
Summarized WSS scores with the taxonomic profile for the FMT donor 18-0002- recipient 18–0018 pair. (A) The WSS scores were determined by comparing 1) the donor’s sample to paired recipient’s pre- and post-FMT samples; and 2) the recipient’s pre-FMT sample to the same recipient’s post-FMT samples for donor 18–0002 and recipient 18–0018. Sample information for this pair was shown in S1 Table. The resultant WSS scores were grouped into different color boxes (see the figure key) or numerical numbers (when the score was below the cutoff value; NS = No Score). (B and C) Relative abundance was obtained for *B*. *uniformis* and *B*. *vulgatus* for this pair using MetaPhlAn2 analysis. For *B*. *vulgatus*, relative abundance for the Recipient was 0.03% and day 21 was 0.05%. For *B*. *uniformis*, relative abundance for the day 42 was 0.02% and day 342 was 0.01%.

An additional donor-recipient pair (donor# 18–0002 and recipient# 18–0018) was then subjected to strain tracking analysis. This FMT was unique in that it had numerous samples that were collected and sequenced for up to 535 days post FMT (Fig 5A). In common with the other four FMTs, all *Alistipes spp*. and *P*. *merdae* were related to the donor for the entire time post FMT. Although the relative abundance of *B*. *uniformis* and *B*. *vulgatus* was found to be higher in donor than recipient pre-FMT, again no consistent pattern was evident between relative abundance and the strains post-FMT (Fig 5B and 5C).

**Fig 6:**
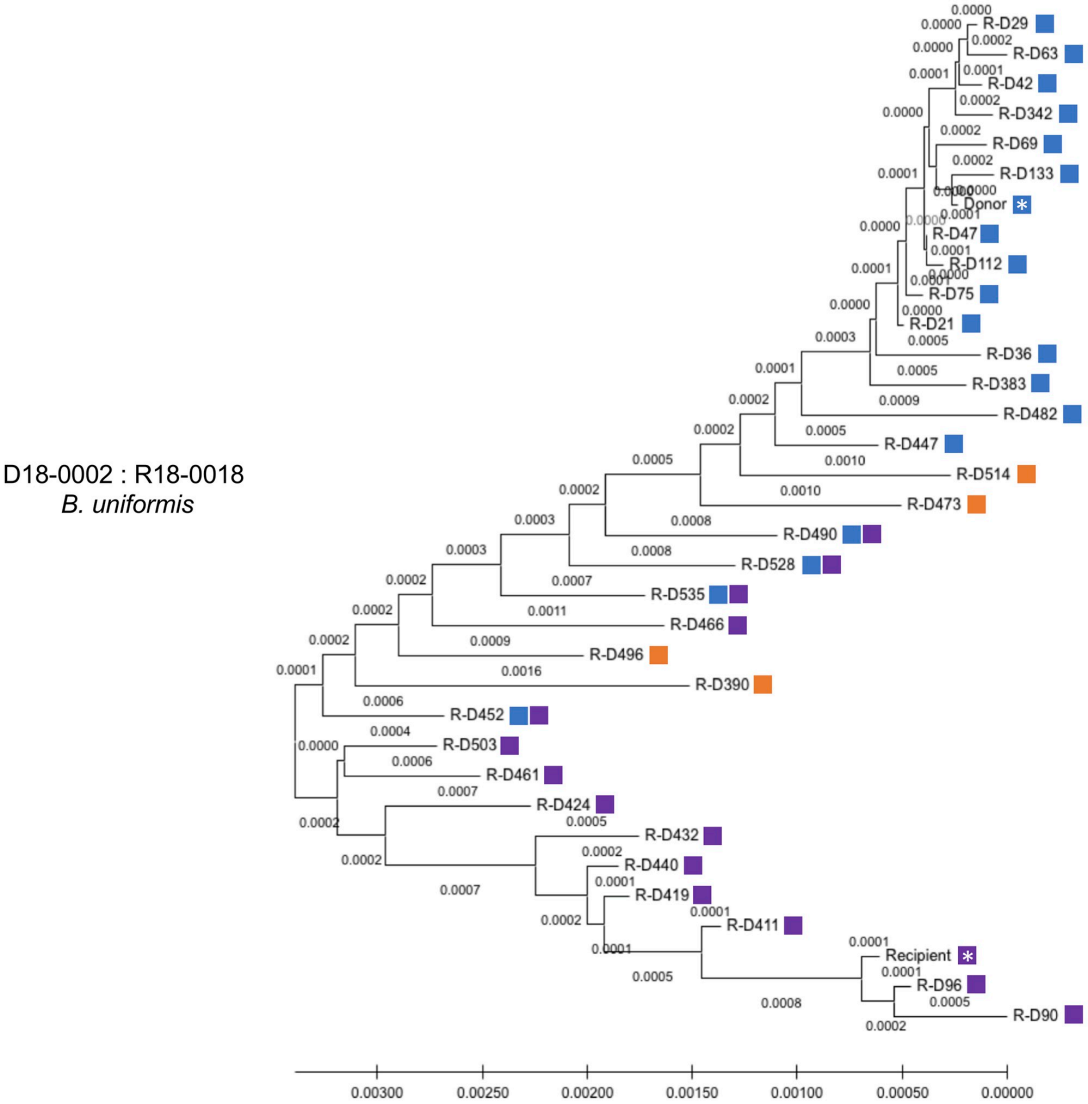
StrainPhlAn analysis for *B*. *uniformis* FMT donor 18–0002 and recipient 18–0018 pair. StrainPhlAn analysis on donor 18–0002 and recipient 18–0018 pair was done for *B*. *uniformis* across all samples in this pair. A neighbor-joining (NJ) tree was constructed, and the tree is drawn to scale with branch lengths. The same steps which written in the legend of Fig 3 were then applied to complete the tree using MEGA X. The blue and purple boxes displayed next to the NJ tree match the color boxes shown in Fig 5A and the orange color boxes shows a recombinant strain which displayed as both numerical numbers in Fig 5A. A white asterisk indicates a donor and recipient pre FMT sample.

The increased number of fecal samples post FMT allowed a more detailed analysis of the strain origin for up to 535 days post FMT. In the case of *B*. *uniformis*, we found that early after FMT, up to approximately 383 days, a pattern of either donor or recipient dominant fecal strains. However, after day 383, we noted several instances where the WSS scores were higher, but below the cutoff value, when compared to both donor and recipient (days 390, 473, 496, and 514). In fact, at some times, the WSS scores were both above the cutoff value indicating relatedness, to both the donor and recipient (days 452, 490, 528, and 535). We used StrainPhlAn to further characterize the relationship of the *B*. *uniformis* strains (Fig 6).

**Fig 7:**
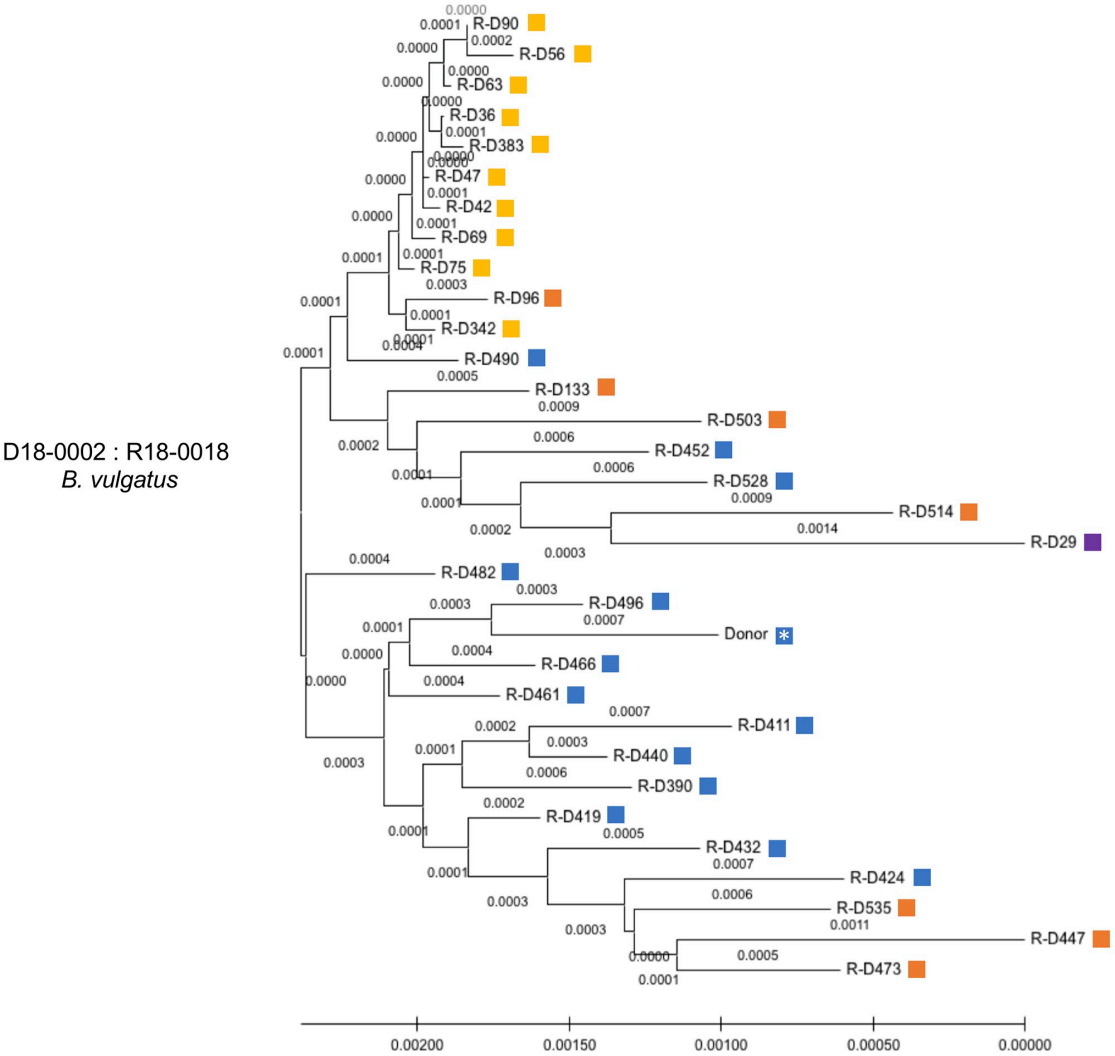
StrainPhlAn analysis for *B*. *vulgatus* FMT donor 18–0002 and recipient 18–0018 pair. StrainPhlAn analysis on donor 18–0002 and recipient 18–0018 pair was done for *Bacteroides vulgatus* across all samples in this pair. A neighbor-joining (NJ) tree was constructed and the tree is drawn to scale with branch lengths. The same steps described in the legend of Fig 3 were then applied to complete the tree using MEGA X. The blue and purple boxes shown next to the NJ tree match the color boxes shown in Fig 5A, the yellow boxes depict a new strain and orange boxes indicate a recombinant strain both shown as numerical numbers in Fig 5A. The yellow boxes showed consistent WSS scores related to donor or recipient and the orange boxes showed either similar WSS scores observed between donor and recipient or a higher score found in the recipient than donor. A white asterisk indicates a donor pre FMT sample.

We found that the donor (blue) and the recipient (purple) were located in distinct clusters on the phylogenetic tree. Importantly, the samples with WSS scores above the cutoff for both donor and recipient were, for the most part, located in branches positioned in between the donor and recipient. A similar result was found for *B*. *vulgatus* where we noted on days 96, 133, 447, 473, 503, and 535 the presence of high WSS for both the donor and recipient (Fig 5A). Analysis of the *B*. *vulgatus* by StrainPhlAn revealed that the majority of the donor (blue) and the recipient (purple (note: only one sample was related to recipient R-D29)) clustered differently. (Fig 7). Within the recipient group (orange), we also found two clusters; one cluster closely related to most of the donor samples (*e*.*g*. R-D 535) and another one related to the recipient (*e*.*g*. R-D514) (Fig 7). Collectively, the results of the strain tracking analysis of *B*. *uniformis* and *B*. *vulgatus* found at later times following FMT the communities of *Bacteroides spp*. were not related to the either the original donor or recipient microbes.

In contrast to the donor-recipient recombinant microbes that were detected in only a few sample time points late after FMT, we did find the *B*. *vulgatus* at days 36, 42, 47, 56, 63, 69, 75, 90 and at two later times, days 342 and 383 had a WSS score of 80–86 compared to the donor and a WSS score of 1–6 compared to the recipient (Fig 5A). In the StrainPhlAn analysis, these samples that had the consistent WSS scores 80–86 related to donor and 1–6 related to recipient had a distinct clustering on the phylogenetic tree from the donor and recipient (Fig 7). Based on the WSS scores and the early emergence after FMT, the origin of this sub-strain is consistent with a minor strain in the donor sample that expanded early after FMT. Thus, the milieu of the recipient post FMT can favor the emergence of a previously undetected microbe sub-strain in the donor.

**Fig 8:**
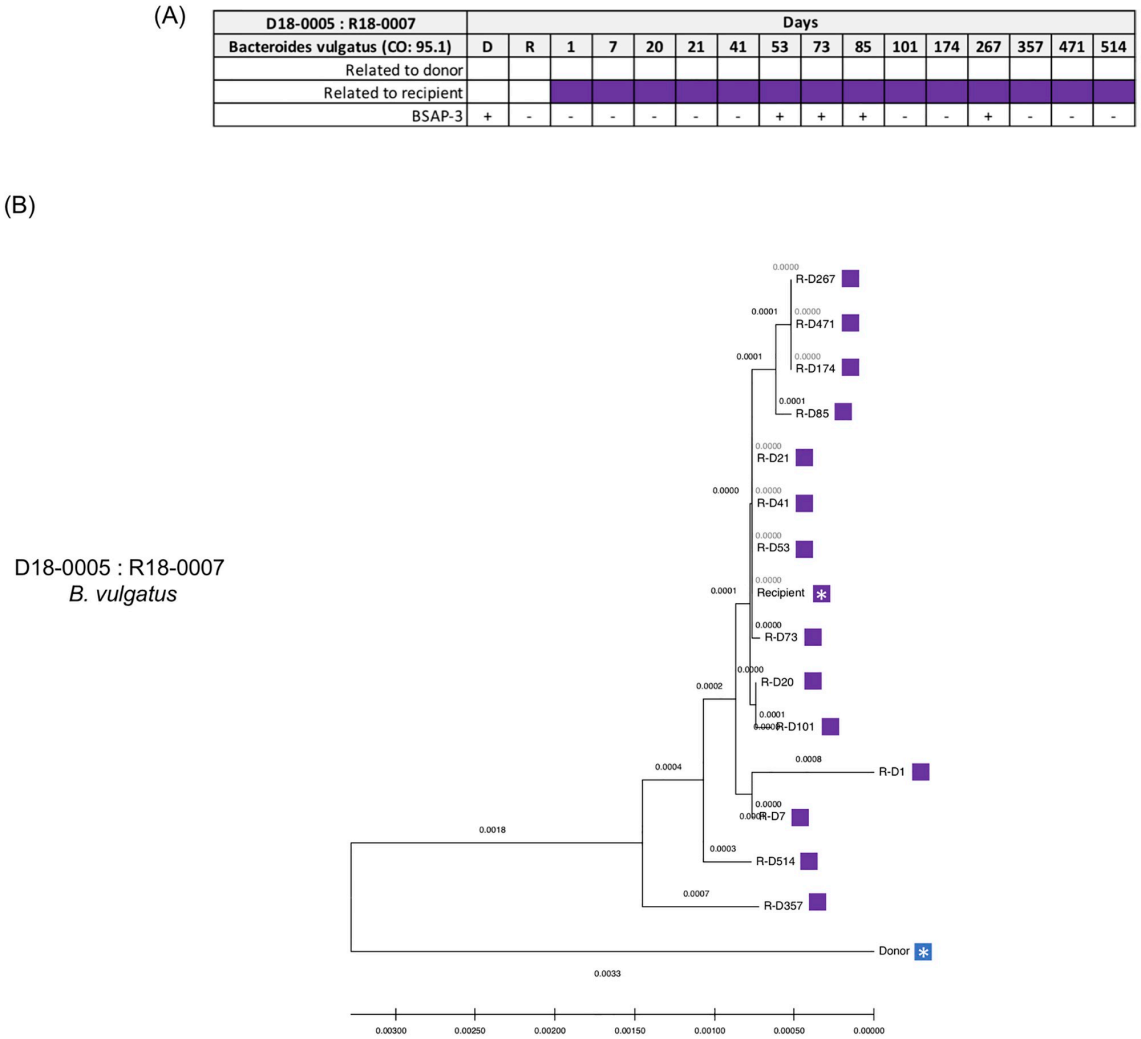
WSS, BSAP-3 and StrainPhlAn analyses for the FMT donor 18–0005 and recipient 18–0007 pair. (A) The WSS scores were obtained by comparing 1) the donor’s sample to paired recipient’s pre- and post-FMT samples; and 2) the recipient’s pre-FMT sample to the same recipient’s post-FMT samples for donor 18–0005 and recipient 18–0007. The purple box indicates that strain was related to the recipient’s pre-FMT. BLAST result from the BSAP-3 gene analysis was also shown in a table, indicating “+” = BSAP-3 positive and “-” = BSAP-3 negative. (B) StrainPhlAn analysis on donor 18–0005 and recipient 18–0007 pair was done for *Bacteroides vulgatus* across all samples in this pair. A neighbor-joining (NJ) tree was constructed and the tree is drawn to scale with branch lengths. The distances were calculated using the Maximum Composite Likelihood method and are in the units of the number of base substitutions per site using MEGA X. The blue and purple boxes shown next to the NJ tree match the color boxes shown in Fig 8A. A white asterisk indicates a donor and recipient pre FMT sample.

In a recent study, we have characterized the HMP data set for the presence of genes encoding *Bacteroidales*-specific antimicrobial proteins (BSAP-3) that have known functions to restrict species specific replication of *Bacteroides vulgatus*. From this analysis, we found that the gene encoding BSAP-3 in the individuals *B*. *vulgatus* were either present or absent [30]. To further characterize the Davar et al. data set in this study, we analyzed the entire data set used in the current study for a gene encoding the complete BSAP-3 protein. In one of the FMT (donor: 18–0005 and recipient: 18–0007) we found that the donor was BSAP-3 positive while the recipient did not have a BSAP-3 gene (e.g. negative) (Fig 8A). Analysis of the recipient post FMT samples for BSAP-3 revealed that four time points (days 53, 73, 85 and 267) had a complete BSAP-3 gene while the remaining samples had incomplete BSAP-3 genes. We confirmed the WSS analysis using StrainPhlAn that the recipient post FMT samples all closely clustered with the recipient (Fig 8B). Thus, for this donor-recipient pair, a recombination event occurred to transfer the BSAP-3 gene to the recipient without changing the relatedness to the original recipient’s dominant fecal strain.

## Discussion

In this study, we have analyzed an extensive time series data set that contains samples for some recipients up to 535 days post FMT. For some donor-recipient post FMT pairs, we found complete fecal dominance of certain donor or recipient microbes (*e*.*g*., *Alistipes spp*., *P*. *merdae*). In contrast, for *Bacteroides spp*. we detected the coexistence of donor and recipient strains for extensive times post FMT. For two FMT pairs (donor 18–0002, recipient 18–0018; donor 18–0014, recipient 19–0024), fecal dominant recombinant strains of donor-recipient were evident post FMT. In addition, FMT pair (donor 18–0002, recipient 18–0018), strain-tracking analysis revealed the presence of a new strain that was most probably a minor strain in the donor feces that emerged following FMT. We also provide evidence for recombination between the donor and recipient in one FMT. Collectively, our results provide a new perspective into the complexity of the microbial strain community in the gut ecosystem following FMT.

A previous study by Li et al. reported the durable presence of donor microbes in the recipients for up to 84 days post FMT in addition to instances where coexistence donor and recipients were detected in the same individual post FMT [16]. In our study, we used a data set with multiple longitudinal samples from 5 independent FMT [17]. A common finding of both studies was that the outcome of FMT with respect to donor colonization of the recipient varied different donor-recipient pairs. We found when the recipient was dominant post FMT there were examples of where the donor microbes were low prior to the FMT (i.e. *P*. *merdae* in Fig 1A; *A*. *putredinis* in Fig 1C; and *A*. *shahii* in Fig 2A) (S2 Table). In contrast, we found when the donor was dominant post FMT there were cases of where the recipient microbes were low prior to the FMT (i.e. *A*. *putredinis*, *A*. *shahii*, and *B*. *uniformis* in Fig 1A; *A*. *onderdonkii* in Fig 1B; and *A*. *onderdonkii*, *A*. *shahii*, and *A*. *putredinis* in Fig 5A) (S2 Table). Most probably, the detection of the donor following FMT would necessitate the colonization of the donor in the available niches of the recipient. The fecal dominance of the donor after FMT indicates that the recipient prior to FMT either did not have the *Alistipes spp*. or *P*. *merdae* or that the amounts were too low to be detected in the feces. In contrast to *Alistipes spp*. and *Parabacteroides*, we found the composition of *B*. *vulgatus* and *B*. *uniformis* fecal strain communities were heterogeneous after the FMT with respect to the dominance of the donor or recipient strain. In some cases, we found the pattern of fecal dominance changed over time in the same individual FMT, suggesting the both donor and recipient strains have coexisted in the niches of the recipient [37].

A unique aspect of the strain tacking analysis of the extensive Davar et al. data set was the identification of potentially new strains of *B*. *vulgatus* and *B*. *uniformis* following FMT from WSS scores that were just below the cutoff for both donor *and* recipient. Using, StrainPhlAn these recombinants showed phylogenetic clustering distinct from the donor and recipient strains. At this point, we cannot distinguish between the simple mixing of individual donor and recipient strains post FMT and the horizontal gene transfer between donor and recipient, which has been demonstrated to occur between strains of *B*. *vulgatus* [38]. However, in the one FMT where a donor that was BSAP-3 was transplanted into a BSAP-3 negative recipient, we did find evidence for recombination of a BSAP-3 gene from the donor to the recipient supporting the possibility of gene recombination between donor and recipient post FMT. Additional studies, beyond the scope of our analysis, would be needed to isolate and grow the microbes from FMT at different times post FMT and use whole genome sequencing to assess the extent of recombination post-transplant.

Finally, from analysis of FMT pairs donor 18–0002 & recipient 18–0018, we found that *B*. *vulgatus* had a unique fecal dominant strain that appeared on days 36–90, and 342–383 that had WSS scores that were similar the donor. In addition, for this strain, the WSS scores were nearly identical over the different analysis days, again suggesting a unique fecal dominant strain that was also supported by StrainPhlAn, which showed a distinct phylogenetic clustering pattern. These features, coupled with the early appearance of this fecal dominant strain after the FMT, suggest that the origin of this strain might have been a sub-strain within the original donor fecal sample that was efficient at colonization in the new host (recipient) and possessed the capacity to initially outcompete both the donor and recipient for fecal dominance. Interestingly, the fecal dominance followed by re-appearance with the same WSS score (again, indicating the same strain) after time supports that this strain effectively colonized the recipient post FMT. The oscillating patterns of fecal dominance of this strain after FMT could reflect differences in the recipient from the donor with respect to the availability of nutrients (e.g. diet) or microbe-microbe and host-microbe interactions in the new ecosystem that transiently favors this strain over the original donor and recipient [15].

## Conclusion

Our time series analysis shows that in FMT where there was no pre-conditioning to reduce or eliminate the recipient microbe community that an individual specific oscillating pattern of donor, recipient and new microbial communities post FMT. The results from our study then provide an important, new framework in which to understand the lack of predictability of FMT to change the gut microbial community function to correspond to that of the donor [16,39]. Although we have only seen this for the *Bacteroides spp*., the mixed donor and recipient microbe communities (and in some instances even new communities of *Bacteroides spp*. recombination), could have a wider impact by altering the interaction between other microbes in the gut ecosystem needed for functions of the gut community in metabolism, colonization resistance and the immune system [12–14]. The practical translation of our analysis suggests the use of pre FMT treatments to reduce recipient microbial communities to facilitate a donor microbial strain dominated gut microbial community following FMT [5,8]. In addition, longitudinal sampling of individual FMT patients in combination with strain tracking analysis to monitor the status of the post FMT microbial community would also be important to assess the stability and ultimately, the success of the FMT [40].

## Supporting information

S1 Table Sample and sequence read information. The original sequence files were sequenced and deposited by Davar et al. in the BioProject database under accession number PRJNA672867. The table represents the sample information with sequence read information used for the analysis in this study.
(XLSX)

S2 Table Taxonomic profile. Microbial community composition was profiled at the species level for (A) donor 18–0002, recipient 18–0018, (B) donor 18–0014, recipient 19–0024, (C) donor 18–0031, recipient 19–0013, (D) donor 18–0006, recipient 19–0002, and (E) donor 18–0005, recipient 18–0007 from Davar data set. The number shown in the table shows the relative abundances obtained using MetaPhlAn2.
(XLSX)
